# Ensemble Algorithm Based on Gene Selection, Data Augmentation, and Boosting Approaches for Ovarian Cancer Classification

**DOI:** 10.3390/diagnostics14242772

**Published:** 2024-12-10

**Authors:** Zne-Jung Lee, Jing-Xun Cai, Liang-Hung Wang, Ming-Ren Yang

**Affiliations:** 1 School of Advanced Manufacturing, Fuzhou University, Quanzhou 362200, China; johnlee@fzu.edu.cn; 2 Graduate School of New Generation Electronic Information Engineer, School of Advanced Manufacturing, Fuzhou University, Quanzhou 362200, China; 3 Department of Microelectronics, College of Physics and Information Engineering, Fuzhou University, Fuzhou 350108, China; 4 Graduate Institute of Biomedical Informatics, College of Medical Science and Technology, Taipei Medical University, Taipei 235, Taiwan

**Keywords:** ovarian cancer, microarray data, gene selection, data augmentation, classification, boosting algorithm

## Abstract

**Background:** Ovarian cancer is a difficult and lethal illness that requires early detection and precise classification for effective therapy. Microarray technology has permitted the simultaneous assessment of hundreds of genes’ expression levels, yielding important insights into the molecular pathways driving ovarian cancer. To reduce computational complexity and improve accuracy, choosing the most likely differential genes to explain the impacts of ovarian cancer is necessary. Medical datasets, including those related to ovarian cancer, are often limited in size due to privacy concerns, data collection challenges, and the rarity of certain conditions. Data augmentation allows researchers to expand the dataset, providing a larger and more diverse set of examples for model training. Recent advances in machine learning and bioinformatics have shown promise in improving ovarian cancer classification based on gene information. **Methods:** In this paper, we present an ensemble algorithm based on gene selection, data augmentation, and boosting approaches for ovarian cancer classification. In the proposed approach, the initial genetic data were first subjected to feature selection. **Results:** The target genes were screened and combined with data augmentation and ensemble boosting algorithms. From the results, the chosen ten genes could accurately classify ovarian cancer at 98.21%. **Conclusions:** We further show that the proposed algorithm based on clustering approaches is effective for real-world ovarian cancer data, with 100% accuracy and strong performance in distinguishing between distinct ovarian cancer subtypes. The proposed algorithm may help doctors identify ovarian cancer patients early and develop individualized treatment plans.

## 1. Introduction

The lifetime risk of developing ovarian cancer is 2.7% among females, representing the sixth highest risk among all types of cancer [1]. The incidence of ovarian cancer is 2.5% of all female cancers; however, 5% of malignant cases result in mortality due to a low survival rate. There is a strong correlation between late-stage diagnosis and the absence of early symptoms [2]. Therefore, the timely identification of ovarian cancer is crucial, given that 70% of women diagnosed with epithelial ovarian carcinoma are only identified once the disease has advanced to the upper abdomen [3].

The application of machine learning, which employs algorithms and data, enables computers to learn and enhance their capabilities autonomously. Its principal strength lies in its capacity to process extensive, intricate, and multi-dimensional data, thereby improving the precision and efficacy of decision making. The potential of machine learning-based approaches for constructing predictive models for platinum reactions in ovarian cancer has been the subject of investigation by researchers. The medical field has witnessed a surge in the adoption of machine learning in recent years, largely due to the growing accessibility of medical data and the continual advancements in computer analysis capabilities [4]. Akazawa and Hashimoto utilized the Extreme Gradient Boosting (XGBoost) machine learning algorithm, achieving an impressive accuracy of 0.80 in classifying ovarian cancer [5]. Zhang et al. demonstrated the effectiveness of deep learning in accurately predicting definitive diagnoses of ovarian cancers using color ultrasound tests [6]. Conversely, only a few studies have incorporated tumor markers or blood parameters. Kawakami’s research revealed that through employing 32 clinical parameters from a blood examination, machine learning could predict diagnoses with an accuracy of 0.92 [7]. Gu et al. employed machine learning to forecast the postprandial change in serum CA125 [8]. Additionally, Lee et al. proposed the use of the most likely differentially expressed genes to circumvent higher computational complexity in the context of ovarian cancer [9]. Machine learning offers the advantage of guiding clinicians and patients in decision making based on ovarian cancer predictions. However, ovarian cancer research often involves the analysis of high-dimensional data, which may be constrained in size, presenting challenges in training effective machine learning models. Consequently, it remains uncertain whether the purported classification accuracy will translate into enhanced performance in clinical settings. Addressing the dimensionality challenge in ovarian cancer research requires a fusion of contemporary machine learning algorithms and a deep understanding of the underlying data characteristics. Efficient feature selection, dimensionality reduction, and data augmentation techniques are pivotal in developing robust and interpretable models for ovarian cancer. Presently, a comprehensive overview of the predictive efficacy of machine learning for classification, incorporating gene selection and data augmentation in the context of ovarian cancer, is lacking. Hence, this study was undertaken to introduce an ensemble algorithm tailored for ovarian cancer analysis. The algorithm under consideration integrates gene selection, data augmentation, and boosting approaches for classification purposes.

The following sections of this work are structured as follows. Section 2 delineates the materials and methods used. Section 3 provides a detailed elaboration of the proposed algorithm. Section 4 presents a comprehensive account of the results and discussions, with a detailed comparison between the proposed algorithm and other existing approaches. Finally, Section 5 presents the conclusions derived from this study.

## 2. Materials and Methods

Ovarian cancer refers to the proliferation of malignant cells in the ovaries, integral components of the female reproductive system. Ovarian cancer can develop from a variety of cell types in the ovaries and has several subtypes, each with unique traits and behaviors. It is staged according to the magnitude of the sickness, and this helps evaluate the cancer’s severity and spread. Patients from China Medical University Hospital provided samples of their ovarian, vaginal, cervical, and myometrium tissues for this study. All microarray operations were carried out in a controlled laboratory setting at the same institution. This study utilized tissue samples from 5 benign ovarian tumors (OVT), 5 ovarian cancers stage I (OVC_I), and 5 stage III ovarian cancers (OVC_III). The pathological and clinical characteristics of OVC_I and OVC_III are detailed in Table 1 [10]. The human cDNA library, comprising 9600 validated human cDNA clones, was provided by the National Health Research Institute of Taiwan. These clones were sourced from the Minimum Information About a Microarray Experiment collaboration libraries through an authorized distributor [11].

**Figure 1:**
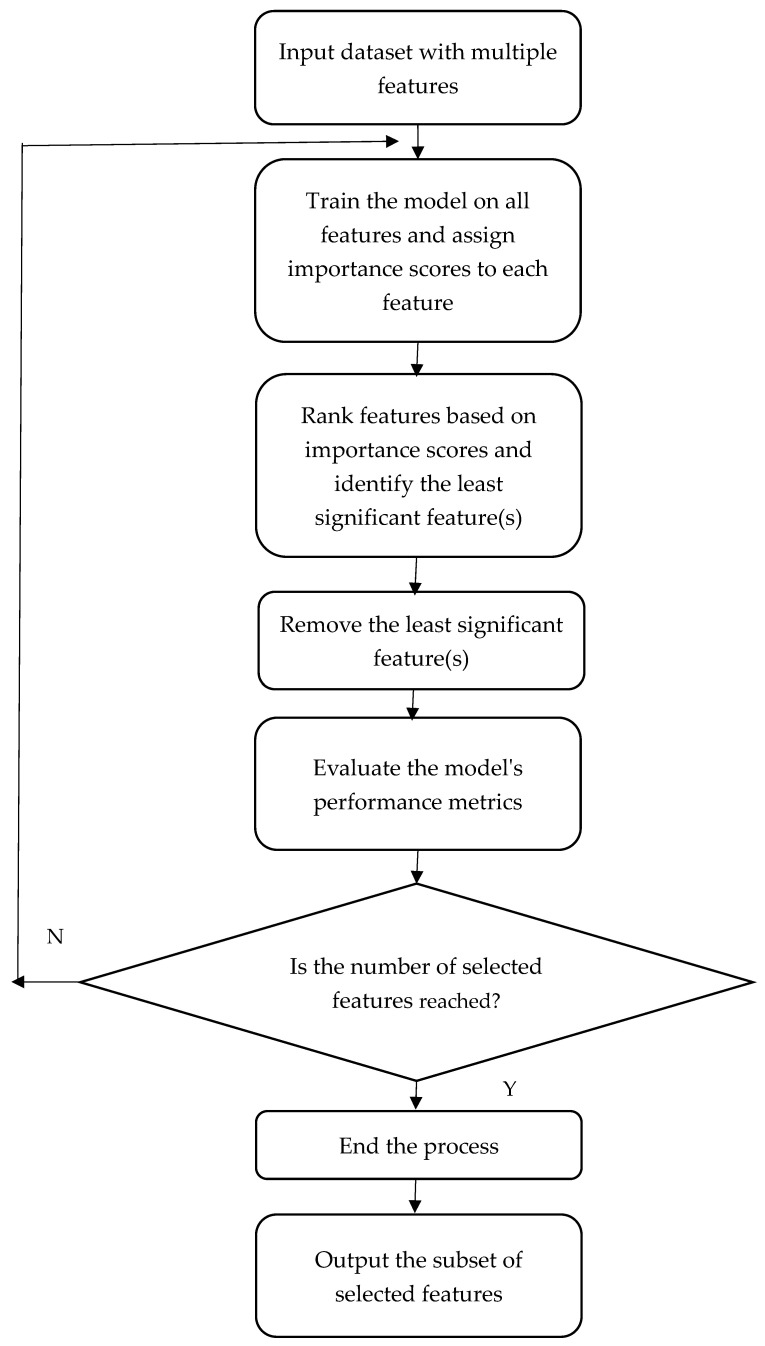
The flowchart of RFE.

In this paper, we introduce an ensemble algorithm that relies on gene selection, data augmentation, and boosting approaches for ovarian cancer classification. Our proposed technique integrates ensemble recursive feature elimination (RFE), data augmentation, and boosting algorithms to identify the most informative genes and enhance classification performance. RFE is a feature selection technique in machine learning. It operates by iteratively removing the least significant features based on model performance to select the most important features. The process involves training the model iteratively and eliminating the least important features until the desired number of features is obtained [12]. RFE works by first training the model on all features and assigning weights to each one based on its relevance. It then removes the least important feature(s) and retrains the model using the remaining features. This technique is continued iteratively until the desired number of features is achieved. RFE rates features according to their impact on the model’s performance. Features with higher ranks are considered more vital, whereas those with lower levels are deemed less relevant. RFE aids in the development of more accurate models that are less prone to overfitting by focusing on the most relevant features. RFE’s chosen subset of features produces a more interpretable model, making it easier to grasp the key reasons driving the predictions. The RFE flowchart is illustrated in Figure 1. The pseudocode is described as follows:

Input dataset with multiple features;
While the number of selected features is not reached, perform the following:
  Train the model on all features and assign importance scores to each feature;
  Rank features based on importance scores and identify the least significant feature(s);
  Remove the least significant feature(s);
  Evaluate the model’s performance metrics;
End the process;
Output the subset of selected features.

**Figure 2:**
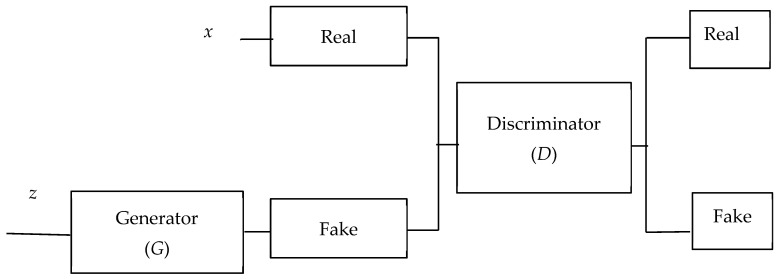
The structure of a GAN.

Data augmentation artificially extends a dataset by generating modified versions of existing data. This technique is frequently utilized in machine learning and deep learning to optimize model performance, particularly in contexts where labeled data are scarce. The process of data augmentation utilizing Generative Adversarial Networks (GANs) entails the exploitation of GAN models to generate synthetic data samples that exhibit a high degree of similarity to the original dataset. A GAN comprises two neural networks, namely a generator and a discriminator, which are trained in an adversarial manner to produce synthetic data that are realistic in appearance. In the context of data augmentation, GANs can be employed to generate supplementary training instances for machine learning models, particularly when the original dataset is limited. After training a GAN on the original dataset, the generator network can learn to produce synthetic data samples that closely resemble authentic data [13]. These synthetic data can then be combined with the actual dataset to provide more diverse instances for training machine learning models. The structure of a GAN is seen in Figure 2.

Boosting is a machine learning technique that aggregates the predictions of multiple weak learners, typically simple models, to create a powerful learner with enhanced predictive capabilities. Boosting algorithms work by sequentially training a sequence of models, with each succeeding model focusing on cases that the prior models misclassified. The final prediction is obtained by averaging the predictions of the individual models. Boosting algorithms, including Gradient Boosting Decision Tree (GBDT), CatBoost, and XGBoost, are well known for their ability to handle complex data relationships while maintaining high predicted accuracy. The foundation of GBDT is the notion that, when it comes to a complicated problem, the consensus reached by a group of experts is superior to the opinion of a single expert. Let the dataset be represented as D=$\left\{(x_{1},y_{1}),\;(x_{2},y_{2}),\cdots ,(x_{n},y_{n})\right\}$. The GBDT algorithm can be seen as an additive model consisting of *m* trees.

(1) $${\hat{y}}_{i}=\sum_{j=1}^{m}f_{j}\left(x_{i}\right),\;f_{j}\in F$$

where *F* represents the function space comprising all trees, with each tree corresponding to a function *f*. In contrast to typical machine learning algorithms, this additive model does not optimize weights in a high-dimensional space; instead, it directly learns a collection of functions. The cost function of the additive model is defined as

(2) $${Cost}^{\left(t\right)}=\sum_{i=1}^{n}loss(y_{i},{\hat{y}}_{i}^{t})+\pi (f_{t})$$

where π represents the complexity of the decision tree, and $loss(y_{i},{\hat{y}}_{i}^{t})$ is the loss function between $y_{i}\;and\;{\hat{y}}_{i}^{t}$ [14]. CatBoost is a gradient boosting framework that utilizes a distinctive tree-based learning algorithm. It is intended to handle categorical features seamlessly and contains built-in support for categorical data [15]. Its fundamental formulation is based on GBDT, a strong machine learning technique that blends gradient boosting and decision tree models. The XGBoost algorithm is a well-known tree-learning technique. Its core component is the second-order Taylor expansion of the cost function, which XGBoost utilizes to ensure model robustness [16]. This algorithm repeatedly splits a new tree to align with the residuals of the previous prediction. By randomly traversing selected features, XGBoost enhances the random sampling ratio and reduces overfitting resulting from examining all features. The development of the XGBoost model is conducted following the aforementioned process. In the initial stage, a preliminary tree is constructed using the training data, and the discrepancy between the anticipated and actual model values is determined. Subsequently, at each iteration, a new tree is constructed to fit the residuals predicted by the model, until the learning process is complete. This iterative process generates a series of residual trees, which are subsequently integrated into diverse tree models.

## 3. The Proposed Algorithm

**Figure 3:**
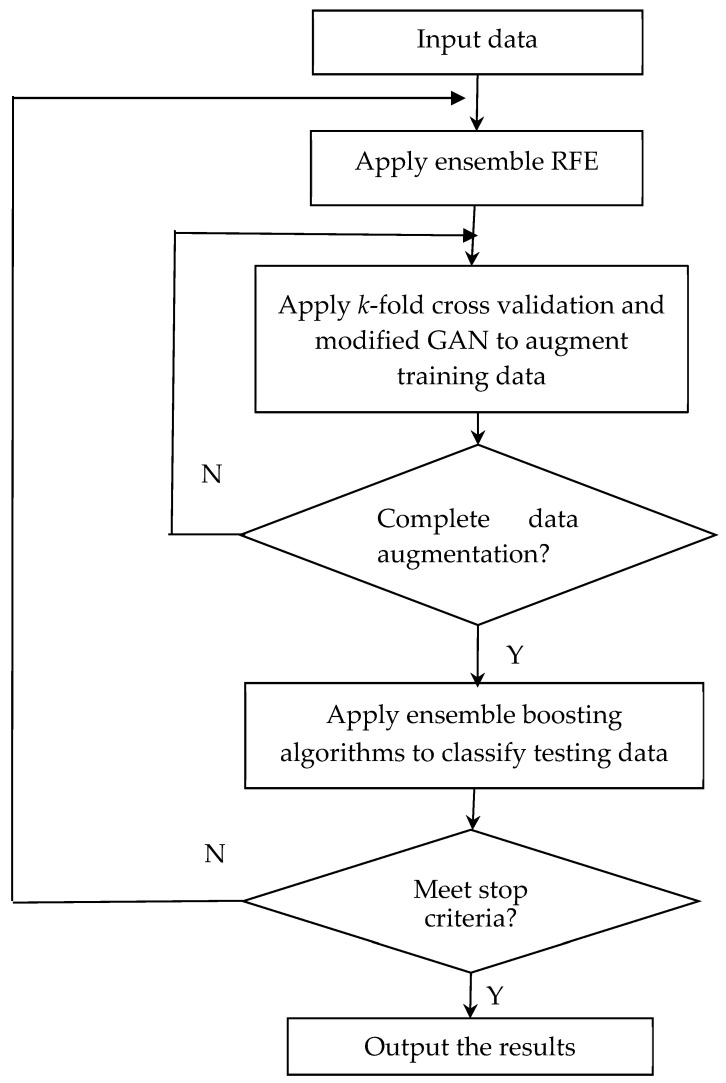
The flowchart of the proposed method.

We applied ensemble RFE, data augmentation, and boosting algorithms to identify the most informative genes and ameliorate classification performance in this paper. The proposed algorithm’s flowchart is depicted in Figure 3. In the proposed algorithm, we apply RFE with GBDT, CatBoost, and XGBoost to select the most important 30 features from 9600 features for each algorithm. This will result in three subsets of features, each selected by a different algorithm. Then, we identify the features that appear in at least two subsets out of the three. These features are potential candidates for the selected features in the ensemble. We select the most important 10 important features from the three subsets to create the ensemble subset of features. These features appear in at least two subsets and are considered important by multiple algorithms.

**Figure 4:**
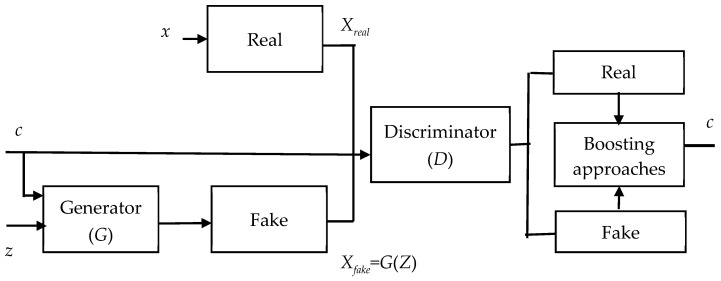
The modified GAN for data augmentation.

Obtaining a vast array of diverse training data for ovarian cancer is not a viable option. Data augmentation is the solution to this limitation. It generates synthetic variations while preserving privacy and security. In this research, we applied a modified GAN to augment data after ensemble RFE. In evaluating the performance of the modified GAN, we employed *k*-fold cross-validation (*k* = 3). This technique partitions the training data into *k* subsets or folds. The model undergoes training and evaluation *k* times, with each iteration utilizing a different fold as the validation set and the remaining *k* − 1 folds as the training set. During each training iteration within the *k*-fold cross-validation process, the modified GAN is trained on the training folds containing the chosen features. It is noteworthy that the generated data are exclusively employed for training purposes. The testing data utilized in this study were derived solely from the original dataset, ensuring that the model’s evaluation and validation were based on authentic, real-world data. Figure 4 illustrates the modified GAN’s ability to produce synthetic data that closely mirror the original training data. It demonstrates the integration of the generative strength of GAN with the discriminative capabilities of boosting algorithms like GBDT, CatBoost, and XGBoost. Data augmentation using GAN and boosting approaches involves generating synthetic data samples that resemble real data instances. The specific modifications applied during the data augmentation process can vary based on the nature of the data and the objectives of the task. Through applying these modifications and validating the generated data through various techniques, data augmentation can effectively enhance the diversity and quantity of the training data, leading to improved model performance and robustness in machine learning tasks. The core network structure of the modified GAN retains a generator and discriminator. The generator takes input comprising both classification details (*c*) and random noise (*z*). In leveraging the boosting algorithm, data classification accuracy is enhanced, effectively tackling the challenge of limited data availability for analysis. In the loss function ${(Loss}_{x})$ of the modified GAN, *x* denotes the raw data. The discriminator’s goal is to maximize ${Loss}_{x}+{Loss}_{c}$, while the generator’s goal is to minimize ${Loss}_{x}+{Loss}_{c}$.

(3) $${Loss}_{x}=E\left[logP\left(x=real|X_{real}\right)\right]+E\left[logP\left(x=fake|X_{fake}\right)\right]$$

(4) $${Loss}_{C}=E\left[logP\left(c=real|X_{real}\right)\right]+E\left[logP\left(c=fake|X_{fake}\right)\right]$$

After training the modified GAN, we created augmented data incorporating the chosen features for every fold within the dataset. This augmented data served to enrich the original training dataset with the selected features, thereby enhancing both the diversity and scale of the training data. Utilizing the augmented training data containing the selected features enabled us to train boosting algorithms on each fold and assess their performance on the respective dataset. Once the *k*-fold cross-validation was complete, we could combine the results from each fold to obtain a clear picture of the model’s overall performance, including the benefits gained from the augmented data generated by the modified GAN. The algorithm continues to run until the stop criteria are satisfied, at which point it produces the final results. By following these steps, we effectively created an ensemble of RFE with GBDT, CatBoost, and XGBoost, and then selected the most important features that appear in multiple subsets. Compared to existing methods, the approach described as the proposed algorithm emphasizes a combination of diversity, feature importance, and cross-validation to identify the most informative and reliable genes for the ensemble. This comprehensive approach aims to address potential limitations of traditional gene selection methods by leveraging the strengths of multiple algorithms, ensuring robustness and enhancing the predictive power of the ensemble model. This approach is the most effective way to combine the strengths of different feature selection algorithms and machine learning models to create a robust ensemble.

## 4. Results and Discussions

To ensure equitable comparisons, maintaining consistent parameters across different methods is crucial. The experiments utilized the *k*-fold cross-validation method with *k* = 3. This research selected 10 features from the proposed algorithm, as shown in Table 2. The target genes were selected using the proposed algorithm, with their cardinalities and F scores all exceeding 10. The gene index of 8683 had the highest F score (19.68) of all the selected genes. These 10 genes were Caenorhabditis elegans, small inducible cytokine A2, KIAA1140 protein, mitochondrial ribosomal protein L23, DKFZp434A115, myxovirus resistance 2, retinoic acid receptor responder 1, protocadherin alpha 5, myeloid differentiation primary response gene, and EGF-containing fibulin.

**Figure 5:**
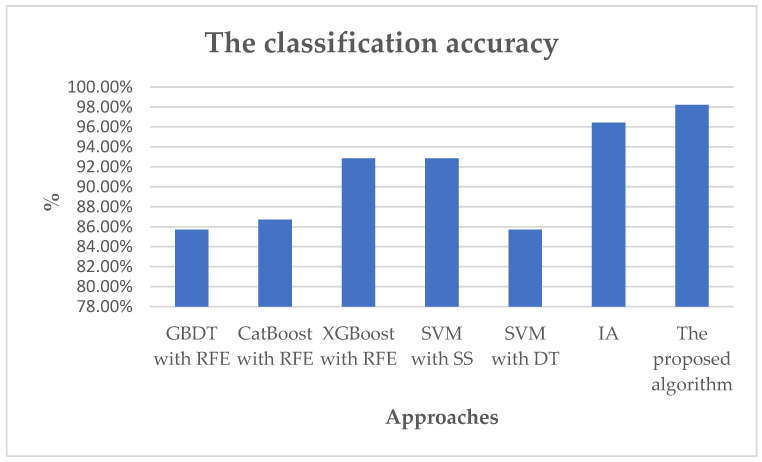
The classification accuracy for various approaches.

A detailed comparison of the various methodologies employed to validate the proposed algorithm’s performance is provided in Figure 5 and Table 3. The results demonstrate that the proposed algorithm enhances the classification accuracy to 98.21% when compared to traditional machine learning techniques, including GBDT, CatBoost, XGBoost, SVM, DT, and IA. The Improved Algorithm (IA) employs a scatter search to identify the optimal parameter configurations for DT. The DT algorithm employs the partition information entropy minimization algorithm to recursively divide the dataset into smaller subsets, which are then represented as tree structures. SVM is a learning algorithm that employs a hypothesis space of linear functions within a high-dimensional feature set. It is also noteworthy that GBDT, CatBoost, and XGBoost incorporate feature selection techniques. The criteria used for feature selection with GBDT is the feature importance, such as for weight, that provides a measure of how each feature contributes to the predictive performance of the model. For CatBoost, the used criteria for feature selection were the prediction value change, which highlights features with higher prediction value change, considering them more significant for the model. As for XGBoost, the used criteria for feature selection were the gain importance, which identify features with higher gain importance, marking them as more influential in the model’s decision-making process.

For index 8683, the gene is weakly similar to a hypothetical protein encoded by the T12A2.1 gene in the caenorhabditis elegans organism. Caenorhabditis elegans is a widely studied model organism in biology, particularly in the field of developmental biology and genetics. Caenorhabditis elegans is a nematode and may seem unrelated to human cancer; it is possible that specific genetic elements or pathways in this organism could have analogs or functional equivalents in human biology, including cancer-related processes [18]. The small inducible cytokine A2 is a protein that in humans is encoded by the CCL2 gene. It plays a crucial role in the recruitment and activation of monocytes, memory T cells, and dendritic cells to sites of injury or infection. The small inducible cytokine A2 plays a crucial role in the regulation of immune responses and inflammation. In the context of ovarian cancer, the presence of specific cytokines may be indicative of tumor microenvironment characteristics, immune cell infiltration, or tumor progression [19]. The KIAA1140 protein is a human protein. The function of the KIAA1140 protein is not yet fully understood, and it is classified as an uncharacterized protein [20]. Mitochondrial ribosomal protein L23 is a protein. It is a component of the large subunit of the mitochondrial ribosome, which is responsible for protein synthesis within the mitochondria, the energy-producing organelles within cells. It plays a crucial role in the structure and function of the mitochondrial ribosome, contributing to the process of translation, where genetic information is used to synthesize proteins [21]. The DKFZp434A115 appears to be a description of a specific mRNA transcript originating from a cDNA clone [22]. Myxovirus resistance 2 is a human protein that is involved in the innate immune response to viral infections. It is particularly recognized for its role in restricting the replication of HIV-1, as well as other retroviruses, by targeting the viral capsid and preventing the nuclear import of viral DNA [23]. Retinoic acid receptor responder 1 is a gene that encodes a protein involved in various cellular processes, including cell growth, differentiation, and apoptosis. It has been implicated in the regulation of cell proliferation and has been suggested to function as a tumor suppressor in certain contexts. The retinoic acid receptor is known to be involved in cell differentiation, apoptosis, and cell cycle control. In ovarian cancer, the dysregulation of retinoic acid signaling pathways could impact tumor cell behavior and response to treatment [24]. Protocadherin alpha 5 is a protein-coding gene that belongs to the protocadherin family, a group of cell adhesion molecules that play key roles in the development and maintenance of the nervous system. These proteins are particularly important in the regulation of neuronal connectivity, including axon guidance, synapse formation, and neuronal circuit assembly [25]. The myeloid differentiation primary response gene is an essential adapter protein involved in the signaling pathways of the innate immune system. It plays a critical role in the transmission of signals from various toll-like receptors and interleukin-1 receptors, which are key components of the innate immune response to pathogens. In the context of ovarian cancer, its expression levels may relate to the tumor microenvironment, immune cell recruitment, and, potentially, response to immunotherapies [26]. EGF-containing fibulin is a gene that encodes a protein involved in the formation and maintenance of the extracellular matrix, a complex network of molecules that provides structural support and influences cellular behavior in various tissues. In ovarian cancer, the dysregulation of genes involved in extracellular matrix interactions can influence tumor invasion and metastasis. It has been implicated in various pathological conditions, including cancer, where its dysregulation has been linked to tumor progression and metastasis in certain types of cancer [27]. Insights from these genes could potentially provide avenues for research in ovarian cancer, such as biological pathways, therapeutic targets, and biomarker development. By delving deeper into the pathways and networks associated with these genes, researchers may gain a better understanding of their roles in ovarian cancer progression and response to therapy. The genes identified in this study hold potential as targets for therapeutic interventions, including targeted therapies and immunotherapies. Furthermore, unraveling the expression patterns and functional significance of these genes could play a pivotal role in the development of prognostic or predictive biomarkers for ovarian cancer.

**Figure 6:**
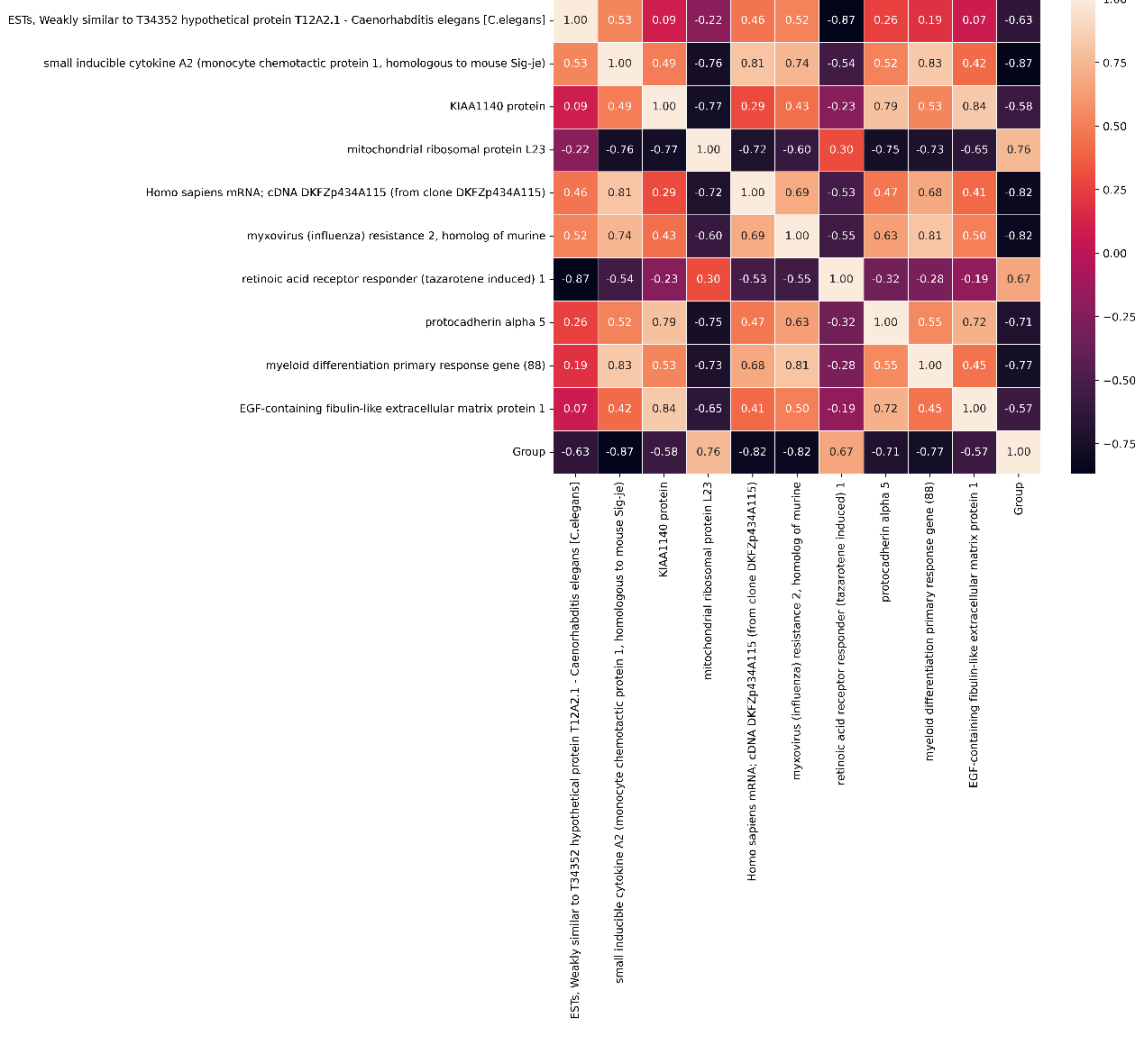
The correlation of the 10 selected target genes.

The correlation of the 10 selected target genes is shown in Figure 6. In Figure 6, the gene of mitochondrial ribosomal protein L23 has the highest positive correlation (0.76) with the label (Group). The dysregulation of mitochondrial function, including alterations in mitochondrial ribosomal proteins, could potentially contribute to cancer development and progression, including ovarian cancer. The gene of small inducible cytokine A2 has the highest negative correlation (−0.87) with the label. Studies have suggested a potential correlation with ovarian cancer, and it may contribute to the vascularization of ovarian tumors.

**Figure 7:**
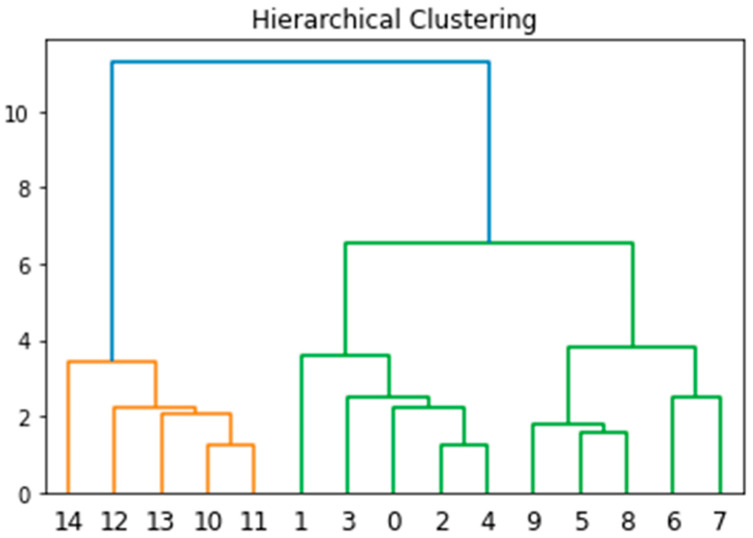
The results of Hierarchical Clustering.

Moreover, these selected genes were verified by Kmeans Clustering, Agglomerative Clustering, and Hierarchical Clustering [28,29]. The simulation results are tabulated in Table 4 and Table 5 and Figure 7. From Table 4 and Table 5, the results show that these gene markers verified by Kmeans Clustering and Hierarchical Clustering can classify OVT, OVC_III, and OVC_I successfully. Figure 7 shows that Hierarchical Clustering has the same performance as Kmeans Clustering. The proposed algorithm demonstrates promising results in classifying ovarian cancer subtypes. The classification model achieved high accuracy and specificity in distinguishing between different cancer subtypes based on gene expression patterns. Comparative analysis with existing methods showed the superior performance of our approach in terms of classification accuracy and robustness.

## 5. Conclusions and Future Work

In this paper, we present an ensemble algorithm based on gene selection, data augmentation, and boosting approaches for ovarian cancer classification. This is the most effective method for analyzing this type of data. The integration of gene selection techniques with ensemble boosting algorithms and data augmentation proved to be an effective method for enhancing the classification of ovarian cancer subtypes. The proposed algorithm leveraging boosting approaches showcases significant performance advantages when contrasted with traditional models like SVM or DT. Boosting approaches, including GBDT, CatBoost, and XGBoost, consistently outperform SVM or DT in terms of classification accuracy. Their effectiveness is particularly pronounced when handling large and intricate datasets, leading to superior accuracy. Furthermore, boosting approaches possess inherent capabilities for capturing intricate non-linear relationships within data, rendering them well suited for addressing a wide array of real-world challenges. In contrast, SVM and DT, while effective in specific scenarios, may encounter difficulties in managing non-linear relationships without the supplemental usage of kernel tricks. Our approach improved the interpretability and predictive power of the classification model by identifying key genes associated with specific cancer subtypes and leveraging the ensemble learning capabilities of the boosting algorithms. These results demonstrate the potential of this intelligent approach to aid clinicians in early diagnosis and personalized treatment strategies for ovarian cancer patients. The outcomes validate the efficacy of this approach in precisely classifying ovarian cancer subtypes and offering significant insights into the fundamental molecular mechanisms of the disease.

To thoroughly evaluate the algorithm’s performance in real-world clinical settings and its ability to generalize to diverse populations, it is imperative to conduct rigorous validation using diverse and representative clinical datasets, accompanied by comprehensive evaluation metrics. Furthermore, assessing the algorithm’s performance across various population groups is crucial for its real-world applicability and ethical deployment. Insights from these selected genes could potentially provide avenues for future research in ovarian cancer, such as biological pathways, therapeutic targets, and biomarker development. By delving deeper into the pathways and networks associated with these genes, researchers may gain a better understanding of their roles in ovarian cancer progression and response to therapy. Moreover, the genes identified in this study hold potential as targets for therapeutic interventions, including targeted therapies and immunotherapies. Furthermore, unraveling the expression patterns and functional significance of these genes could play a pivotal role in the development of prognostic or predictive biomarkers for ovarian cancer. Exploring the integration of multi-omics data, including genomics, transcriptomics, proteomics, and metabolomics, could enrich the model’s understanding of the molecular landscape of ovarian cancer. This integration may provide a more comprehensive view of the disease and potentially lead to the discovery of novel biomarkers or therapeutic targets.

## Figures and Tables

**Table 1:** The pathological and clinical characteristics of OVC_III and OVC_I.

Age	Pathology	Stage
44	Mucinous cystadenoma and borderline malignancy	OVC_I
39	Serous cystadenoma and borderline malignancy	OVC_I
46	Mucinous cystadenoma and borderline malignancy	OVC_I
58	Clear cell carcinoma	OVC_I
36	Mucinous cystadenoma and borderline malignancy	OVC_I
26	Mucinous cystadenoma and borderline malignancy	OVC_III
51	Serous cystadenocarcinoma	OVC_III
62	Serous cystadenocarcinoma	OVC_III
53	Clear cell carcinoma and endometrioid adenocarcinoma	OVC_III
61	Serous cystadenocarcinoma	OVC_III

**Table 2:** The 10 selected genes from the proposed algorithm.

Index	Gene Name	F Score
8683	Caenorhabditis elegans	19.68
6760	Small inducible cytokine A2	18.17
1720	KIAA1140 protein	16.32
3958	Mitochondrial ribosomal protein L23	15.09
6269	DKFZp434A115	12.90
5954	Myxovirus resistance 2	12.85
3699	Retinoic acid receptor responder 1	12.84
4201	Protocadherin alpha 5	12.10
6324	Myeloid differentiation primary response gene	11.28
3390	EGF-containing fibulin	10.97

**Table 3:** The classification accuracy for ovarian cancer.

Method	Classification Accuracy	Selected Genes
GBDT with RFE	85.71%	30
CatBoost with RFE	86.71%	30
XGBoost with RFE	92.86%	30
SVM with SS [17]	92.86%	12
SVM with DT [17]	85.71%	14
IA [17]	96.43%	5
The proposed algorithm	98.21%	10

**Table 4:** The stage results of Kmeans Clustering and Agglomerative Clustering with 10 selected genes.

Stage	Kmeans Clustering #	Agglomerative Clustering #
OVT	0	0
OVT	0	0
OVT	0	0
OVT	0	0
OVT	0	0
OVC_IA	1	1
OVC_IA	1	1
OVC_IA	1	1
OVC_IC	1	1
OVC_IA	1	1
OVC_IIIB	2	2
OVC_IIIC	2	2
OVC_IIIC	2	2
OVC_IIIC	2	2
OVC_IIIC	2	2

**Table 5:** The clustering results of Kmeans Clustering and Agglomerative Clustering with 10 selected genes.

Cluster #	OVT	OVC				Total
		IA	IC	IIIB	IIIC	
0	5	0	0	0	0	5
1	0	4	1	0	0	5
2	0	0	0	4	1	5
Total	5	4	1	4	1	15

## Data Availability

The utilized code and data will be supplied upon request with the approval of the healthcare facility.
